# Greening Infection Prevention and Control: Multifaceted Approaches to a Sustainable Future

**DOI:** 10.1093/ofid/ofae371

**Published:** 2024-07-03

**Authors:** Pamela S Lee, Irene Frantzis, Shira R Abeles

**Affiliations:** Division of Infectious Diseases, Department of Medicine, Harbor-UCLA Medical Center, Torrance, California, USA; Department of Pediatrics, Columbia University Irving Medical Center, New York, New York, USA; Department of Infection Prevention and Control, NewYork-Presbyterian Hospital, New York, New York, USA; Division of Infectious Diseases and Global Public Health, Department of Medicine, University of California, San Diego, San Diego, California, USA

**Keywords:** climate change, environmental sustainability, hospital epidemiology, infection prevention and control, pollution

## Abstract

Infection prevention and control, or IP&C, is a critical stakeholder in advancing environmental sustainability in health care. IP&C activities seek to ensure safety of processes in health care from an infection perspective, but how these are performed can drive substantial waste and pollution. There are certain IP&C measures that can, without compromising safety or efficiency, be adapted to more environmentally friendly practices and have a high impact benefit to sustainability without affecting patient outcomes. Moreover, IP&C practice stands to be significantly altered by climate change and pollution. Here, we describe the complex interdependence between sustainability, climate change, and IP&C, and opportunities for IP&C to be at the leading edge of optimizing healthcare's environmental footprint.

## HEALTH CARE AND INFECTION PREVENTION AND CONTROL AS CONTRIBUTORS TO CLIMATE CHANGE AND POLLUTION

Global warming secondary to greenhouse gases and pollution from human activity have reached historic highs and continue to surge. Climate effects are increasingly harming nature, wildlife, the global economy, society, and human health [1]. Pollution is estimated to be responsible for 9 million premature deaths, the majority from low- and middle-income countries, as well as minority and marginalized populations regardless of location [2].

Climate change and pollution are projected to cause an additional 14.5 million deaths by 2050, with severe damage to our global economy and health care systems [3]. Yet the health care system itself is a major contributor to global emissions [4]. The US health care system leads the globe with the highest per-capita greenhouse gas emissions from health care [5]. In 2018, US health care greenhouse gas emissions composed 8.5% of total national emissions and health care-associated air pollution and emissions were estimated to cause the loss of 388 000 disability-adjusted life years in the United States alone [5]. In response to this health care-derived morbidity and mortality, the World Health Organization released a call for response to the climate change challenge, emphasizing the need to build more climate-resilient and environmentally sustainable health systems [6].

The field of infection prevention and control, or IP&C, aims to prevent infections in health care delivery settings. In the United States, hospital IP&C became established in the 1950s because of nosocomial outbreaks of *Staphylococcus aureus* [7]. These outbreaks demonstrated a clear need to protect the public from health care-acquired infections and led to the creation of IP&C departments. The initial goals of IP&C were described as surveillance, control, and education—goals that IP&C continues to fulfill to this day despite mounting challenges such as antimicrobial-resistant organisms and novel pathogen pandemics. Modern-day IP&C departments are responsible for extensive activities, which strive toward the overarching goal of maintaining a safe and sanitary environment for the provision of healthcare. IP&C activities are substantial and varied, including oversight of isolation precautions, contact tracing, mandated reporting to national data repositories on health care-associated infections, and ensuring health care systems processes such as aseptic techniques, device insertion/maintenance, and hand hygiene are followed. Unfortunately, how some of these IP&C activities are operationalized creates significant health care waste inadvertently because of factors such as the prevalence of single-use disposable medical devices and use of disposable personal protective equipment (PPE).

## CLIMATE CHANGE AND PLASTIC POLLUTION POSE A THREAT TO IP&C

IP&C practice has the potential to be dramatically impacted by climate change. Climate change and environmental factors ultimately impact the health of whole communities and contribute to infectious complications that IP&C is beholden to prevent. Pathogen adaptation in response to climate change has significant infectious disease, including IP&C, implications [8–10]. For example, *Candida auris*, a multidrug resistant yeast and threat within health care facilities worldwide [11] has spread even in the COVID-19 era with strict isolation protocols [12, 13]. This pathogenic yeast has at least in part become a new virulent organism as a result of climate change and the acquisition of thermotolerance in a warming world [14]. Ironically *C auris* identification in health care settings prompts aggressive IP&C measures that generate significant pollution and carbon emissions including use of PPE for contact precautions, terminal disinfection, and use of single-use disposable devices [15, 16].

Environmental plastic pollution may complicate IP&C by promoting antimicrobial resistance. The health care system uses a tremendous amount of plastic, estimated at about 3000 tons of plastic waste each day in the US health care system alone [17]. Plastics, although convenient, are increasingly identified as harmful to human health [18, 19]. They contain many chemicals of concern, and importantly they do not biodegrade and thus persist in the environment where they erode into microplastics and nanoplastics. Microplastics may exacerbate antimicrobial resistance given their persistence in the environment where they serve as substrates for pathogenic bacteria and biofilm formation [20, 21]. Because hospitals are concentrated sites of antimicrobial and disinfectant use, and now plastic as well, hospital wastewater in particular may serve to incubate antimicrobial resistance further [22]. Indeed, hospital water systems can be reservoirs of resistant Gram-negative pathogens that likely need system replacement for effective decolonization [23, 24].

## HARMONIZING THE PRIORITIES OF IP&C AND HEALTH CARE SUSTAINABILITY

IP&C is a key player in effecting sustainable change across the health care spectrum—from generating facility-specific policies to advocating for environmentally favorable regulatory guidelines at the state and national level. Next, we describe opportunities for IP&C and health care sustainability to collaborate and highlight select high-level barriers to prioritizing sustainability within IP&C (Table 1).

**Table 1.** Proposed Pathways for Enacting Sustainable Change in IP&C and Select Barriers to These Pathways

Scope of Care	Opportunity for Sustainable Change	Barriers to Sustainable Change
Direct patient care	Individualized risk assessment for transmission-based precautions	Facility-level buy-in Regulatory oversight
	Diagnostic stewardship	Clinician awareness
Health care infrastructure	Environmental controls in procedure and operating rooms	Health care facility capacity for environmental controls
	Increasing local use of reusable devices and PPE	Availability of adequate sterile reprocessing facilities Space for storing devices Staffing for reprocessing
	Optimizing waste management	Improper waste sorting
Health care ecosystem	Increasing widespread availability of reusable devices/PPE	Restrictive instructions for use Product availability
	Minimizing non-evidence-based and resource-wasteful practices	Regulatory oversight

Abbreviations: IP&C, infection prevention and control; PPE, personal protective equipment.

## SUSTAINABLE IP&C OPPORTUNITIES IN PATIENT CARE

### Transmission-based Precautions—Individualized Risk Assessment

IP&C departments oversee the employment of transmission-based precautions to prevent spread of multidrug resistant organisms (MDROs). These precautions may be recommended by a regulatory body but policies are individualized at the facility level, and practices are highly variable across institutions. For example, the utility of contact precautions for methicillin-resistant *Staphylococcus aureus* (MRSA) and vancomycin-resistant *Enterococcus* (VRE) is debated in IP&C [25]. Multiple studies suggest that contact precautions do not reliably reduce endemic MRSA or VRE spread [25–27]. Given these findings, a considerable number of hospitals long ago abandoned contact precautions for MRSA/VRE [25, 28, 29], but use of contact precautions for these MDROs remains prevalent in health care [25]. Contact precautions typically employs use of disposable PPE with single use gowns and gloves, which confer a significant environmental impact [30, 31]. On an inpatient general medicine ward 56 kg of single-use PPE were disposed of during a 24-hour period, more than one-third of all municipal solid waste collected from the ward during that time [32]. Because up to 25% of hospitalized patients may be in contact precautions for MRSA or VRE [25], decreasing unnecessary contact precautions for these organisms may have a major impact on health care's carbon footprint. Moreover, contact precautions use has other deleterious consequences: it has been associated with increased frequency of preventable adverse events including falls and pressure ulcers [33, 34], fewer provider visits [25], and increased hospital cost [35].

Health care epidemiologists and infection preventionists can perform facility-level individualized risk assessments for MDRO transmission to determine when and how to employ contact precautions and other transmission-based precautions [36]. IP&C departments can work with sustainability professionals to advocate for strong horizontal IP&C practices such as high adherence to hand hygiene, rather than waste-generating practices such as contact precautions—an effort that serves both IP&C and sustainability. IP&C can also support efforts to transition to more environmentally favorable PPE, such as reusable cloth isolation gowns. Reusable cloth isolation gowns have been shown to decrease greenhouse gas emissions, decrease water consumption, and decrease waste in comparison to disposable gowns [37]. Properly laundered and processed reusable health care textiles, including reusable PPE, appear to have minimal risk of health care-associated infection [38]. Reusable cloth PPE has also been shown to build resilience within health care systems, as was demonstrated with supply chain shortages during the COVID-19 pandemic [39]. Employing reusable PPE allows organizations to continue to use PPE when necessary while also reducing environmental impact and conferring other key benefits. Innovations in biodegradable and recyclable PPE as alternatives to single-use disposable are an area of active research. Further studies regarding the efficacy of these biodegradable and recyclable products, their environmental impacts, and how they can be integrated into health care delivery processes are required.

### Diagnostic Stewardship

IP&C and sustainability initiatives overlap when it comes to optimizing patient care in diagnostic stewardship. Infection preventionists dedicate significant effort to supporting diagnostic algorithms to avoid unnecessary microbiological testing and subsequent overdiagnosis. For example, reducing overdiagnosis and misdiagnosis of *Clostridioides difficile* mitigates cascading consequences for patients and facilities [40]. Patients may be colonized with *C difficile*, but if subjected to molecular testing when not indicated, they risk being misdiagnosed with *C difficile* infection. This results in wasted testing supplies, wasted PPE from unneeded contact isolation, and unnecessary antibiotics. Misdiagnosis causes worse patient care and reflects inaccurate hospital-onset *C difficile* rates [41]. Similarly, avoiding overtesting of urine and identifying asymptomatic bacteriuria (ASB), particularly among persons with urinary catheters or admitted from long-term care facilities, has co-benefits across many areas of patient care including IP&C, antimicrobial stewardship, and sustainability. It has been estimated that more than 45% of ASB identified results in needless antimicrobial use [42]. In a single-center pilot study, better stewarded urine testing could have potentially saved several tons of plastic waste each year [43]. Reducing unnecessary testing of ASB can reduce waste from testing supplies and avoidance of unnecessary antibiotic therapy.

Preventing true hospital-acquired infections has direct benefits to patients and health care institutions in reducing further resource consumption [44]. Avoiding central line infections, catheter-associated urinary tract infections, *C difficile* infections, ventilator-associated events, surgical site infections, and other health care-associated infections reduces morbidity, mortality, intensive care unit stays, and length of stay [45]. Preventing health care-associated infections also prevents significant downstream waste, as every day in a hospital is estimated to be associated with 29 pounds of waste [46]. Preventing extended length of stay and stays at higher levels of care therefore in and of itself advances sustainability and health simultaneously.

## SUSTAINABLE IP&C OPPORTUNITIES IN HEALTH CARE INFRASTRUCTURE

### Environmental Controls

IP&C can also collaborate and guide health care infrastructure toward more environmentally conscientious, but still patient-centered, practices. Environmental controls in health care settings are an important aspect of IP&C, particularly in operating rooms, and are some of the most energy-intensive aspects of a health care facility [47]. These heating, ventilation, air conditioning, and air filtration practices are key aspects of infection prevention to mitigate spread of respiratory infections, and in the operating rooms, to decrease bioburden of the air. Given their high energy requirements, they must be reassessed with a planetary health lens. Interventions such as reducing the high-energy heating, ventilation, and air conditioning requirements of operating rooms during off hours can significantly reduce energy requirements without compromising air quality measurements [47–49]. IP&C can collaborate with facilities engineering in collaborative efforts to reduce energy consumption and maintain patient safety where operationally feasible.

### Optimizing Waste Management

US hospitals produce more than 5.9 million tons of waste each year [50], including 1.7 million tons of plastic waste [51]. Hospital waste is segregated into different disposal streams that require different levels of processing. Biohazardous waste confers a dramatically higher environmental footprint compared to nonbiohazardous waste because high-energy processes such as autoclaving or incineration are frequently required before terminal disposal [52]. Unfortunately, waste audits have repeatedly demonstrated that nonbiohazardous waste is frequently sorted into biohazardous bins, generating unnecessary greenhouse gas emissions and increasing health care costs [53, 54].

Effective waste management in health care facilities requires collaboration between multiple services including environmental services, facilities management, and IP&C. Interventions to address improper sorting of biohazardous waste are a common and effective project to improve sustainability in health care [55, 56]. Collaboration between sustainability professionals and IP&C on waste management projects is a clear synergistic opportunity for health care sustainability and IP&C, which will both reduce greenhouse gas emissions and provide important data on waste disposal streams and composition.

## BARRIERS TO SUSTAINABILITY EFFORTS IN IP&C

### Challenges With Regulatory Requirements

IP&C is a highly regulated field, wherein infection preventionists are expected to maintain standards issued by local, state, and national agencies. Regulatory oversight from these organizations is critical to formalize and institute IP&C processes. However, these regulatory requirements may drive low-yield practices, and may lag behind new evidence and serve as barriers to sustainability goals. For example, as tuberculosis rates in the United States have declined, testing recommendations for US health care workers have adapted from mandatory annual testing to becoming more targeted and selective while remaining data-driven [57]. But while tuberculosis testing evolved appropriately, it remains a regulatory requirement for healthcare facilities to perform yearly “fit” testing for respirators for patient-facing employees, though there is strong evidence that this is a low-yield, high-cost exercise that also unnecessarily uses numerable N-95 masks [58]. Similarly, certain local or state agencies may also require health care facilities to perform surveillance testing for select MDROs. As MDRO epidemiology shifts—ie, previously rare/emerging organisms become endemic—regulatory requirements should shift in tandem as further data on the risk of transmission becomes available. Infection preventionists are well-positioned to identify these low-yield, burdensome, and ultimately wasteful requirements, but it requires regulatory agencies to be engaged in updating requirements to promote optimal practices and decrease health care waste at the same time.

### Widespread Use of Single-use Devices

There has been a significant movement toward single-use device utilization in medical care, from fairly simple devices such as single-use blood pressure cuffs, to high complexity procedural instruments such as bronchoscopes, laryngoscopes, endoscopes, and other surgical supplies [59–61]. Although single-use items offer convenience and a sense of being indisputably microbe-free, they persist and cause harm after their single use in the health care setting. Moving back toward reusable items in health care will significantly reduce greenhouse gas emissions and waste and ultimately benefit planetary health. Such a movement will also necessitate coordination and investment across the health system. Adoption of reusable devices requires that manufacturers generate products designed for reuse, procurement invests in reusable products, and that health care facilities have the infrastructure and staff in place to effectively clean devices to prevent patient harm.

Industry-set “instructions for use,” or IFUs, determine the regulatory requirements guiding the use and potential reuse of medical devices. Simple, noncritical medical devices are frequently designed as single-use, disposable items. Some of these noninvasive and noncritical medical supplies, such as single-use blood pressure cuffs, have been branded as important to IP&C by manufacturing companies despite existing IP&C guidance for safely cleaning reusable forms of the products [62]. Other items may not exist yet in a cost-effective, practical form that meets IP&C and sustainability goals. For example, scissors used for nonsterile activities such as cutting gauze or tape, would be most practically designed for reuse with wiping with disinfectant [63]. Unfortunately, the only available scissors for purchase may be labeled as single use, or as reusable with IFUs that require sterilization, which is impractical. If reusable medical grade scissors with practical IFUs are not available, practice is driven to single-use scissors and enforced by regulatory requirements.

Disposable high-complexity devices provide convenience as they do not require the space, time, trained staff, and equipment for high-level disinfection (HLD) or sterilization. During regulatory surveys, one of the top “not compliant” findings among health care institutions is in sterilization and disinfection processes [64], further hindering widespread adoption of reusables. Moreover, certain complex devices such as endoscopes have been linked to serious health care-associated infections from MDROs [65, 66], further incentivizing the implementation of single-use versions. Single-use versus reusable endoscopes and colonoscopes is an area of great interest and study, with current opinions advocating for likely a mixed use of reusable and disposable scopes, depending on practice type, procedure requirements, and resources dedicated to sterilization and HLD to both optimize safety, reliability, and sustainability [67]. However, both the financial cost and environmental footprint of disposables are higher than reusables among high-volume centers [68–71]. Sterilization and HLD may also expose workers to harmful chemicals such as ethylene oxide, which has carcinogenic and other hazardous health properties [72]. Thus innovation promoting sustainable design, improving reliability of disinfection, and optimizing the longevity of instruments is necessary to meet both sustainability and safety goals.

Partnerships with key stakeholders such as device manufacturers, supply chain, regulatory bodies, and device operators are needed to help shepherd in optimally designed reusable devices and help move practices toward a circular economy. IP&C should be involved early in facility design to ensure that clinics, operating rooms, procedural areas, and sterile processing facilities are set up to allow for cost-effective, reliable, and sustainable practices that benefit patient and planetary health. Furthermore, IP&C can partner with regulatory bodies such as the Food and Drug Administration to streamline processes for developing reusable items, advocate for the inclusion of environmental impact data such as lifecycle assessments in product design and provide flexibility in IFU oversight. Such efforts can propel IP&C forward as a thought leader in informing evidence-based regulatory requirements that prioritize both planetary and patient outcomes.

## CONCLUSION

IP&C is positioned to be a tremendous ally for improving the environmental footprint of health care. The tradeoff with systemic harm to public health must be considered in the calculation of the methods used to prevent infections today. Multidisciplinary efforts, with commitments from the IP&C community, are necessary to overcome current barriers to reducing health care's environmental impact. IP&C can guide systems to smart design to minimize risk to patients now and in the future. Engaged health care epidemiologists and infection preventionists can identify collaborative projects where sustainability can be improved without placing patients or staff at infectious risk. Furthermore, IP&C practitioners can work with organizations such as the Centers for Disease Control and Prevention, the Joint Commission, the Food and Drug Administration, the Society for Healthcare Epidemiology of America, and the Association for Professionals in Infection Control and Epidemiology to develop IP&C policies that carry forward the decades-old ideals of IP&C while also prioritizing planetary health.

## Notes

***Financial support***. The authors declare no sources of funding for this paper.

***Patient consent statement.*** There are no factors necessitating patient consent.
